# Diverse Clinical and Histology Presentation in C1q Nephropathy

**DOI:** 10.5812/numonthly.8308

**Published:** 2013-06-25

**Authors:** Pavan Malleshappa, Mahesha Vankalakunti

**Affiliations:** 1 Division of Nephrology, Department of Medicine, Adichunchanagiri Institute of Medical Sciences, Mandya, India; 2 Department of Nephro-pathology, Manipal Hospital, Bangalore, India

**Keywords:** Kidney Diseases, Proteinuria, Therapeutics

## Abstract

Patients presenting with nephrotic syndrome with or without nephritic illness rarely come across with the diagnosis of ‘C1q nephropathy’. This entity is purely diagnosed with the help of immunofluorescence like IgA nephropathy. Clinical presentation is heterogenous, ranging from nephrotic range proteinuria to sub-nephrotic state; and with or without hematuria / renal insufficiency. Similarly, the concept of ‘C1q nephroapthy’ has periodically evolved since its original description by Jenette and Hipp in 1985. Here the pathophysiology, histologic findings / diagnostic and therapeutic options in patients with C1q nephropathy are discussed.

## 1. Introduction

C1q nephropathy refers to a disorder in which C1q deposits are seen in mesangium on immunofluorescence microscopy and mesangial electron dense deposits on electron microscopy (1, 2). C1q nephropathy (C1qN) was first described by Jenette and Hipp in 1985 (1). They proposed the distinct clinical entity of C1qN with the diagnostic features of (1) lack of clinical and serological evidence of SLE with the (2) presence of dominant or codominant deposition of C1q in mesangium on immunofluorescence. The prevalence of C1q nephropathy varies from 0.2 to 16.0% and seems to be higher in children (2, 3). C1q nephropathy usually presents with proteinuria or full-blown nephrotic syndrome. This entity refers to a pattern of glomerular injury based on varying histopathologic findings, including no glomerular lesions to focal segmental glomerulosclerosis (FSGS), and proliferative glomerulonephritis (4). C1q nephropathy is a rare glomerulonephritis with a varied natural history, which makes it difficult to conduct studies on treatment. Thus, the optimal treatment of C1q nephropathy is not clearly defined. Corticosteroids have been tried with mixed results, with most of the studies showing poor response to oral corticosteroid therapy (2).

The outcomes of patients with C1q nephropathy can be predicted by a variety of clinical and histologic variables. Favourable outcomes in patients with C1q nephropathy are associated with lower levels of proteinuria, nephritic syndrome and histologic variant of minimal change disease. Unfavourable outcomes may be predicted by nephrotic range proteinuria and FSGS variant of C1q nephropathy (2).

This review discussed the pathophysiology, histologic findings/diagnostic and therapeutic options in patients with C1q nephropathy.

## 2. C1q – Key Factor in Complement Activation and Source

C1 is the first component of the classical complement pathway. C1 is a pentamer composed of five molecules: a single C1q, two C1r, and two C1s. The classical activation pathway is initiated by the binding of C1q to immune complexes. This binding causes a distortion in the C1q, which in turn activates C1r and C1s. Activated C1s cleaves C4 and thereby initiates the classical complement cascade. C1q is a 400kDa protein. C1q comprises 6 A, 6 B and 6 C chains, each possessing a globular N-terminal domain and a conserved C-terminal region (5-7). C1q binds strongly to IgM, IgG1 and IgG3, but binds weakly to other immunoglobulins, such as IgG2, and does not bind at all to IgG4, IgA, IgD and IgE. C1q first binds to the Fc portion of antigen-bound IgG or IgM after which C1r and C1s attach to form C1, the first enzyme of the pathway. The activated C1 enzymatically cleaves C4 into C4a and C4b. The C4b then binds to adjacent proteins and carbohydrates on the surface of the antigen and then binds C2. The activated C1 cleaves C2 into C2a and C2b forming C4b2a, the C3 convertase. Thus the classical complement pathway is activated.

The role of C1q is not restricted to recognition of immune complexes. C1q is a key player in placental development, onset of preeclampsia, regulation of autoimmune diseases such systemic lupus erythematosus and plays a critical role in the pathogenesis of prostate cancer.

C1q is synthesized extrahepatically by a wide range of cell types including monocytes/macrophages, epithelial cells, mesenchymal cells, dendritic cells, trophoblasts, microglial cells, fibroblasts, and endothelial cells. The synthesized C1q is expressed as a cell membrane associated molecular ligand. The C1q receptors are expressed on monocytes, macrophages, polymorphonuclear cells, fibroblasts, platelets, lymphocytes, endothelial cells and mesangial cells. C1q receptors play a role in enhancing binding of immune complexes to human mesangial cells (8).

## 3. Pathogenesis

The pathophysiological mechanism by which C1q deposition is likely to cause disease has remained enigmatic necessitating further studies to explore inciting factors of C1q nephropathy. Possible theories include: a) Binding of C1q to the trapped immunoglobulins. C1q deposition in mesangium may be as a result of binding to Fc portion of IgM and IgG either via direct interactions with surface-bound Ig or via trapping of circulating immune complexes. Considering this mechanism, some authors postulated C1q nephropathy could represent as a variant of FSGS (3). b) Contrary to this theory, presence of mesangial electron-dense deposits argues against the disease mediated by podocytes injury, hence away from the spectrum of FSGS (9). c) There are reports depicting causal relationship between C1q nephropathy and viral infections, especially DNA viruses (BK Polyoma virus (10) and Epstein-Barr virus (11). d) Rarely, abnormalities in C1q inhibitor protein may pose risk factor for deposition of C1q (12).

## 4. Histologic Patterns of C1q Nephropathy

### 4.1. Light Microscopy

**Figure 1.**
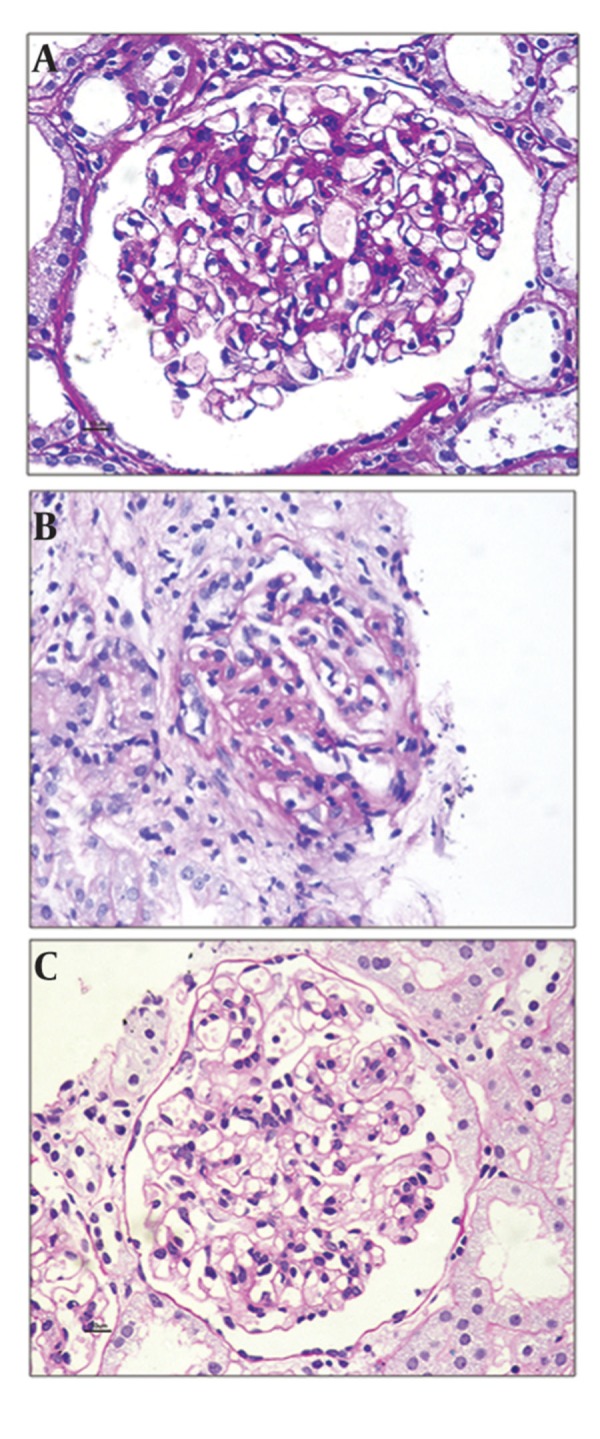
A) Minimal change disease - like morphology in C1q nephropathy. B) Focal and segmental glomerulosclerosis in C1q nephropathy. C) Mild degree of mesangial cell proliferation affecting globally in C1q nephropathy.

The histologic patterns of C1q nephropathy could be divided broadly as (Figure 1): A] Minimal change disease (MCD) (Figure 1a), B] Focal segmental glomerulosclerosis (FSGS) (Figure 1b), and C] Immune mediated proliferative glomerulonephritis (GN) (Figure 1c). Latter group encompasses different morphologic appearances ranging from focal/diffuse mesangial proliferative GN, post infectious GN, membranoproliferative GN, and membranous GN. The first report of Jenette et al. in 1985 comprised 15 patients, revealing MCD in 2 cases, mesangialhypercellularity in 3 cases and focal /or diffuse proliferative GN in 8 cases (1). Markowitz et al. in 2003 reported predominant histology as FSGS in 17 cases and MCD in 2 cases. The recently published report on the largest study group correlating clinicopathologic changes of C1q nephropathy (n = 72) describe FSGS in 11 (16%), MCD in 27 (38%), proliferative glomerulonephritis in 20 (28%), and a variable picture in other patients, i.e., tubulointerstitial nephritis and thin basement membrane nephropathy (13). Occasional case report of crescentic glomerulonephritis is seen in C1q nephropathy (14).

### 4.2. Immunofluorescence Microscopy

**Figure 2.**
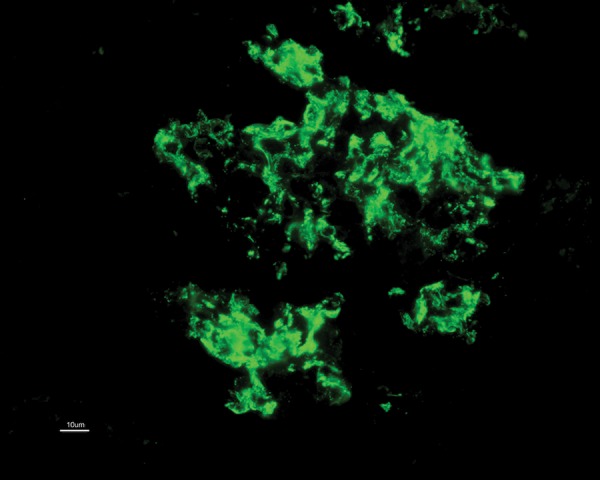
Intense Mesangial Deposition of C1q (3+)

C1q nephropathy is based on demonstration of intense C1q (dominant or co-dominant) positivity, mainly in the mesangium (Figure 2). As a component of immune complexes, IgG and IgM serve as ligand for binding of C1q and further activation of classic pathway of complement cascade. Hence other immunoglobulins could also be positive; however they are ≤ intense as C1q. The frequency of positivity for IgG, IgA, IgM, C3 and C4 is 66%, 34%, 80%, 83% and 35% respectively (13). One of the largest study groups (n = 72) on C1q nephropathy, full house pattern is documented in upto 30 % of them with dominant or co-dominant C1q expression. Having said that multiple immunoglobulins can be positive, there is a chance that a patient can in fact fulfill the diagnostic criteria for both C1q nephropathy and IgA nephropathy. The latter combination could be avoided to a greater extent as C3 staining is seen more in cases of IgA nephropathy (15).

### 4.3. Electron Microscopy

Electron dense deposits in C1q nephropathy are confirmatory of the diagnostic entity. Irrespective of light microscopic wide spectrum, the deposits are always seen in mesangium. In addition to the mesangial location, deposits can also be seen in subendothelial and subepithelial area in cases of morphologic appearances of proliferative glomerulonephritis or focal segmental glomerulosclerosis. ‘podocytopathy’ as evidenced by minimal change disease/focal segmental glomerulosclerosis in light microscopy, possess podocytes foot process effacement and cytoskeleton condensation to a wider extent (13).

## 5. Clinical Presentation

The prevalence of C1q nephropathy ranges from 0.2 to 2.5% in biopsies from children and adults, to 2.1 and 6% in pediatric biopsies, to 16.5% among renal biopsies of children with nephrotic syndrome and persistent proteinuria (13). It generally affects older children and young adults, with an average age of 17.8 years, with an equal gender distribution. Two variants of C1q nephropathy are describted: MCD/FSGS and immune-complex mediated glomerulonephritis variant. Immune complex GN variant includes mesangial proliferative glomerulonephritis, membranous nephropathy and membranoproliferative-like glomerulonephritis. Secondary C1q nephropathy may be seen in patients with viral infection or rarely with rheumatoid arthritis (16). There are few case reports wherein the patients had presented with rapidly progressive glomerulonephritis (17) as well as acute renal failure requiring hemodialysis (18). C1q nephropathy may present as the nephrotic syndrome, the nephritic syndrome or isolated proteinuria/hematuria (12). Hypertension is present in about 50% of patients. Renal insufficiency at the time of diagnosis is quite frequent.

Majority of patients present with persistent proteinuria. Spontaneous remission is uncommon but has been reported (19). Patients with the FSGS variant of C1q nephropathy often show a poor response to treatment. Renal insufficiency is eventually seen in majority of cases and 3 year renal survival is about 80% (20).

## 6. Management and Response

There are no randomized trials that have evaluated the treatment of C1q nephropathy. Current therapy involves treatment of the underlying light microscopic lesion. Glucocorticoids remain the mainstay of treatment. Most of the studies have indicated poor response to glucocorticoids (1, 3). Methylprednisolone pulse therapy has shown to be effective in steroid resistant cases. Sequential therapy with cyclophosphamide, azathioprine, mycophenolatemofetil, tacrolimus and rituximab used separately or in combination with steroids has shown good clinical response in different studies. Since its initial description there have been numerous published case series and several case reports of patients with this condition.

Tanja Kersnik Levart et al. followed up 12 children with C1q nephropathy (20). Eight out of 12 patients presented with nephrotic syndrome. Only one of these, a patient with MCD, responded excellently to corticosteroid therapy and experienced no relapse during the 1-year follow-up period. Four patients, three with MCD and one with FSGS associated with DMP, became corticosteroid dependent, but responded very well to cyclophosphamide. Three patients with MCD experienced complete remission, while in one patient with FSGS associated with DMP, partial remission was achieved. The remaining three patients with nephrotic syndrome and FSGS, two of them with associated DMP, were corticosteroid resistant and unfortunately also showed very poor response to other immunosuppressive therapy (cyclophosphamide, cyclosporine A, chlorambucil and mycophenolatemofetil). One of them progressed to end-stage renal failure, one had transitory acute renal failure, while in the last patient the follow-up was too short (6 months) to predict the final outcome. In addition, end-stage renal failure was observed in one patient with FSGS, who presented with nephrotic proteinuria and renal insufficiency.

Alenka Vizjak et al. followed up 53 patients with C1q nephropathy, for 4 months to 21 years (mean 5.4 ± 5.1) (13). A kidney donor with no light microscopy lesions retained normal kidney function after 15 years. Among five patients with asymptomatic hematuria and/or proteinuria and no light microscopy lesions, one had complete remission, one had partial remission, and three had stable renal disease after 6 months to 7.5 years without treatment. All 13 patients with nephrotic syndrome and minimal change like lesion and eight of nine with nephrotic syndrome and FSGS received corticosteroids (10 received sequential therapy with cyclophosphamide including one who also received cyclosporine, one cyclosporine and mycophenolatemofetil, and one azathioprine, tacrolimus, and mycophenolatemofetil; one received sequential therapy with mycophenolatemofetil; and one with cyclosporine). The majority (76.9%) of the minimal change like group but only one third (33.3%) of the FSGS group were in complete remission after 4 months to 21 years, and four patients had partial remission after 4 months to 3 years. One patient with FSGS had resistant nephrotic syndrome despite 3 years of combined immunosuppressive therapy, and three (33.3%) had end-stage renal disease (ESRD) 2.5, 4.0, and 9.0 years after biopsy. Among 14 patients with proliferative glomerulonephritis, only four received immunosuppressive therapy. Two were treated with cyclophosphamide and had complete remission after 1.5 and 4.0 years. One patient received corticosteroids and had stable renal disease after 3.0 years. One patient, treated with corticosteroids and azathioprine, had stable renal disease after 2.0 years. Among 10 untreated patients, six had stable renal disease after 0.5 to 6.5 years, two had progressive renal disease after 12.0 years, and two had ESRD after 5.5 and 7.0 years.

Satoshi Hisano et al. followed up 61 patients with C1q nephropathy. According to presentation at onset, patients were divided into two groups: asymptomatic urinary abnormalities (asymptomatic) (n = 36) and nephrotic syndrome (NS) (n = 25) (21). Nine of 10 patients in the asymptomatic group and all patients in the NS group were treated with prednisolone and/or cyclosporine. Normal urinalysis was found in 10 patients in asymptomatic group and 8 in NS group during the follow-up. Thirteen patients in the NS group were frequent relapsers at the latest follow-up. Three patients with FSGS developed chronic renal failure 8 to 15 years after the diagnosis. C1q deposits disappeared in 3 of 8 patients receiving repeat biopsy, and 2 of these 3 showed FSGS. They concluded that in long-term follow up, the prognosis of C1q nephropathy is good.

Rituximab, an anti-CD20 antibody has been tried in two patients who had failed to respond to immunosuppressive therapy. One of them achieved normalization of renal function; and hemodialysis was eliminated in the other patient (22).

On the contrary, relevance of C1q-dominant deposition in allograft is addressed by Said et al. in 2010 in a series of 24 patients. None of them were diagnosed as C1q nephropathy in the native renal biopsy or had any features of systemic lupus erythematosus. Mesangial deposits of C1q were detected in up to 82 % of the cases, and it was usually detected after the first year of transplantation. They concluded that C1q-dominant mesangial deposition in the renal allograft is a morphological pattern with no apparent clinical significance in majority of the patients (23).

In summary, a trial of glucocorticoids similar to that described for treatment of FSGS may be tried. A better response to this therapy would be expected in those with the minimal change lesion than those with an underlying lesion of FSGS.

## 7. Conclusion

C1q nephropathy may be an under-recognized entity, and is found in patients with a wide clinical and histological spectrum. Further studies are needed to define the range of its histological and clinical features, treatment options and prognosis. Routine use of C1q staining of renal biopsy analysis is recommended.
